# Restorative perception of urban streets: Interpretation using deep learning and MGWR models

**DOI:** 10.3389/fpubh.2023.1141630

**Published:** 2023-03-30

**Authors:** Xin Han, Lei Wang, Jie He, Taeyeol Jung

**Affiliations:** ^1^Department of Landscape Architecture, Kyungpook National University, Daegu, Republic of Korea; ^2^School of Architecture, Tianjin University, Tianjin, China; ^3^School of Architecture, Harbin Institute of Technology, Shenzhen, Shenzhen, China

**Keywords:** deep learning, street view, semantic segmentation, multiscale geographically weighted regression, urban street environment, restorative quality

## Abstract

Restorative environments help people recover from mental fatigue and negative emotional and physical reactions to stress. Excellent restorative environments in urban streets help people focus and improve their daily behavioral performance, allowing them to regain efficient information processing skills and cognitive levels. High-density urban spaces create obstacles in resident interactions with the natural environment. For urban residents, the restorative function of the urban space is more important than that of the natural environment in the suburbs. An urban street is a spatial carrier used by residents on a daily basis; thus, the urban street has considerable practical value in terms of improving the urban environment to have effective restorative function. Thus, in this study, we explored a method to determine the perceived restorability of urban streets using street view data, deep learning models, and the Ordinary Least Squares (OLS), the multiscale geographically weighted regression (MGWR) model. We performed an empirical study in the Nanshan District of Shenzhen, China. Nanshan District is a typical high-density city area in China with a large population and limited urban resources. Using the street view images of the study area, a deep learning scoring model was developed, the SegNet algorithm was introduced to segment and classify the visual street elements, and a random forest algorithm based on the restorative factor scale was employed to evaluate the restorative perception of urban streets. In this study, spatial heterogeneity could be observed in the restorative perception data, and the MGWR models yielded higher *R*^2^ interpretation strength in terms of processing the urban street restorative data compared to the ordinary least squares and geographically weighted regression (GWR) models. The MGWR model is a regression model that uses different bandwidths for different visual street elements, thereby allowing additional detailed observation of the extent and relevance of the impact of different elements on restorative perception. Our research also supports the exploration of the size of areas where heterogeneity exists in space for each visual street element. We believe that our results can help develop informed design guidelines to enhance street restorative and help professionals develop targeted design improvement concepts based on the restorative nature of the urban street.

## 1. Introduction

Streets are used as passageways to a city; however, streets can be considered symbols of civilization, from which we derive multiple social functions, e.g., culture, politics, democracy, economy, and health (1). Compared to other urban spaces, e.g., squares and green spaces, city parks, and community parks, urban streets are functionally more diverse and complex, and they are important places for urban residents to walk, communicate, shop, exercise, and participate in multiple social activities (2). As the urbanization process continues, multiple negative impacts occur, e.g., urban sprawl, environmental damage in the central city, and the decline of street vitality. When people stay in a low-quality urban environment for a long time period, they will feel stress, and prolonged serious stress can to lead to mental illness and even physical illness (3). People born in urban areas possibly suffer from mental illness than those born in rural areas; as people become more urbanized and live longer, their risk of illness increases (4). However, high-quality urban streets are restorative and invigorating, and they can reduce psychological stress, clear negative emotions, and effectively reduce the incidence of many chronic diseases (5). The restorative nature of urban streets indicates that they can help people reduce stress and various negative emotions, relieve mental fatigue, and achieve improved mental and physical health (6). Hartig stated that “restoration” refers to regaining physical, psychological, and social abilities lost in the process adapting to the external environment (7). The restorative nature of high-quality urban streets and the reduction of psychological stress have led researchers and urban planners to focus on the creation of high-quality restorative urban streets (8).

Early restorative environmental research focused on natural environments or urban environments dominated by natural elements (9). For example, Nordh examined restorative perception by selecting 74 images of a park and identified the restorative characteristics of a park environment (10). With the expansion of scientific perspectives in recent years, the restorative potential of urban streets is being gradually explored. As one of the most important public spaces in cities, the restorative nature of urban streets should be carefully considered. Researchers in many fields have attempted to understand the restorative effects of urban streets. For example, Linda et al. studied the restorative nature of urban streets and reported that tall buildings around urban streets hinder the perception of restorative nature. Furthermore, Lindal and Hartig reported that plants along streets have a positive effect on the perception of restorative nature (11). Zhang et al. used virtual reality technology to simulate an urban street environment and reported that plants along the street demonstrated positive correlation with restorative perception (12). Although such methods can help us understand the restorative perception of urban streets, they are limited in terms of the restorative perception measurement of large-scale complex streets. Time-consuming, small sample size, high cost, and low efficiency can only be studied for restorative perception of small-scale sites, and not for large-scale street perception. Recently, because of the development of multi-source big data services, e.g., Baidu Street View (BSVI), Tencent Street View (TSVI), and Google Street View (GSVI), high-quality and reliable research data are available to examine the restorative perception of urban streets on a large scale. Furthermore, with the rapid development of computer technology, the application of deep learning is becoming increasingly widespread, and more researchers are employing deep learning technologies to solve urban problems (13). The use of multiple linear regression models to explore the relationship between the perceptions of cities and multiple explanatory variables is well established (14); However, many studies have been conducted to model and analyze the cause and effect for the global space. Street view images with geographic coordinates are standard spatial data, research on the restorative perceptions of street views may be heterogeneous in space, and global spatial regression interpretation analysis of spatial data may have errors, which can lead to biased results that are not accurately representative of reality. The GWR and MGWR models are more accurate for spatial data because they take into account geospatial heterogeneity and make use of local regression. Thus, the choice of a suitable spatial processing model is important for the accurate representation of the results (15, 16).

The study area considered in this study is the Nanshan District of Shenzhen City, Guangdong Province, China, which is covered by Baidu Maps compared to other map systems (17). Using Baidu Maps enables a more detailed study of urban streets in the Nanshan District; thus, Baidu Street View was used as a data source for the restorative perception of urban streets. This study has two primary objectives. (1) Prediction of restorative perception of urban streets using a random forest algorithm based on a restorative scale to identify the characteristics of visual element combinations in urban streets under different restorative perception scores. (2) The effect of visual street elements on restorative effects is identified using OLS, GWR, and MGWR to select the model having the most explanatory power. Compared with a single global regression model, a comprehensive study using OLS, GWR, and MGWR models can determine whether the restorative perception data are smooth in space and whether a spatial regression model needs to be selected for the study, and secondly, comparing different spatial regression models, the model with the highest accuracy can be selected for the phenomenological interpretation, which can more accurately reflect different regions of different visual elements in space for impact on restorative perception. This has an important practical significance for rational regional planning. The joint analysis method of street view big data, deep learning, and the spatial regression model can help urban planners and urban humanities researchers to have more targeted and deeper understanding and knowledge of the restorative perception of urban streets.

## 2. Workflow, study area, and data collection

**Figure 1:**
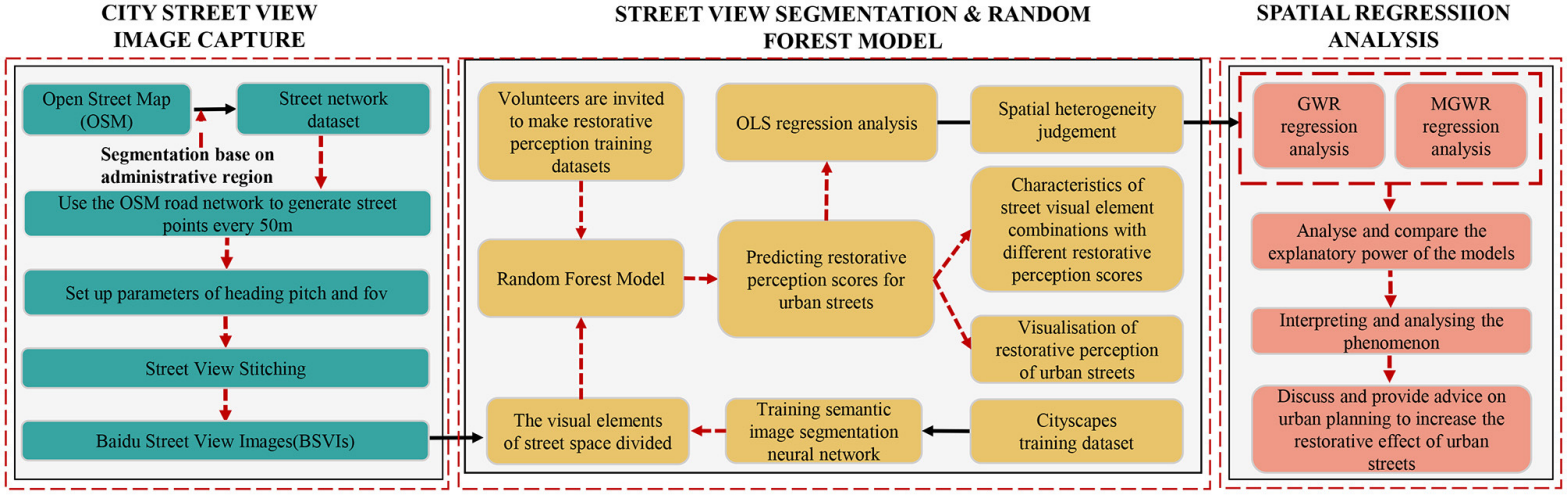
Overview of our workflow.

Figure 1 shows the conceptual framework of urban street restorative perception exploration from a holistic perspective. Firstly, the road network of the study area is downloaded from the OSM website based on the administrative area, a street spot is taken every 50 m of the road network, and the street view image acquisition parameters are adjusted by simulating the pedestrian's viewpoint, and the Baidu Map API is called to collect the urban street view big data, and four street view images of the same street spot are stitched together to finally obtain all the street view images of the study area. The semantic segmentation of the training images is used to obtain the data of urban street view visual elements, while volunteers are invited to score some street views for restorative perception according to the restorative perception scale, and the scoring results and the street view visual elements are made into a machine learning training dataset and imported into a random forest model to predict the restorative perception of all the street view images in the study area. The restorative perception scores were subjected to OLS regression analysis with the visual elements of urban streets, and the results were judged to be spatially heterogeneous, and then GWR and MGWR regression analyses were conducted to select models with high explanatory strength for the restorative perception phenomenon, and finally to discuss and provide urban planners' opinions on improving the effect of restorative perception.

### 2.1. Study area

**Figure 2:**
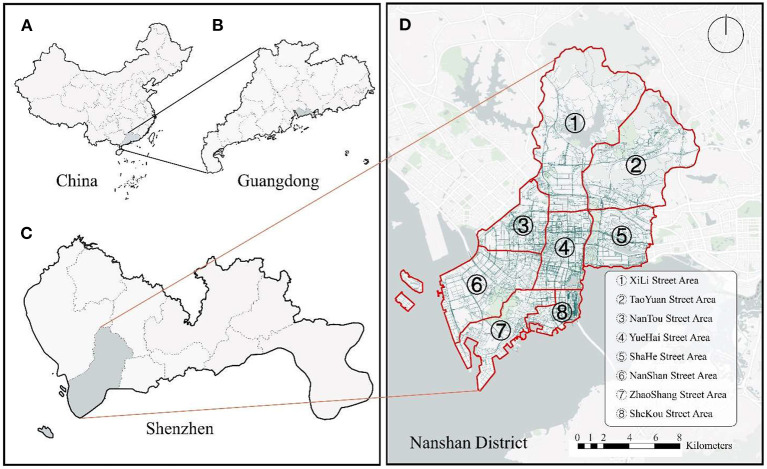
Study area (Nanshan District, Shenzhen, China): **(A)** China; **(B)** Guangdong Province; **(C)** Shenzhen; **(D)** Nanshan District.

In this study, we focused on one of the most economically developed regions in China, i.e., the Nanshan District, Shenzhen, Guangdong Province (Figure 2). The population of Nanshan District is ~1.79 million, accounting for 10.23% of the resident population of Shenzhen. The Nanshan District has several hills, the highest of which is 587 meters above sea level, several bays (including Shenzhen Bay), five islands, and numerous rivers and reservoirs. Nanshan District is bordered by the ocean and has a coastline of 43.7 km. The geographical and environmental conditions are very rich compared to other regions; thus, the potential perceptual spatial heterogeneity caused by the geographical environment can be fully considered. In addition, Nanshan District is the center of scientific research, education, and sports in Shenzhen, and is the location of Shenzhen University, Southern University of Science and Technology, Shenzhen High-Tech Industrial Park, and the Shenzhen Bay Sports Center. As one of the faster-growing economic regions in Shenzhen, Nanshan District is likely more advanced in terms of urban construction and renovation. Empirical research on restorative perceptions in Nanshan District can serve as a test for other developing regions and provide suggestions to guide how other regions can improve restorative perceptions on the path to urbanization. Thus, Nanshan District was selected in this study to investigate the perceived restorative nature of urban streets.

### 2.2. Collecting Baidu street view image data

Street view images were collected along roads in the Nanshan District to mimic human perspective in order to assess the restorative perception of the area. Urban environment analysis studies that use streetscape image big data to evaluate urban street quality are becoming increasingly common (18). Street view data can be used to perceive and observe an urban environment from a human perspective (19). The street view data platform provides street view browsing services to web users and publishes an application programming interface (API) that allows users to download massive amounts of street view data. Thus, street view data are used to represent the environment of city streets as a basis to measure restorative perception. To collect street view images, street view collection georeferenced points were generated at 50-m intervals using the roadway network provided by OpenStreetMap. Then, the Baidu Map API interface was used to download the latest street view image data in 0°, 90°, 180°, and 270° views at each point location. Specifically, each coordinate point is collected the 360° image of the street view, and each point consists of 4 images (0–90°, 90–180°, 180–270°, 270–360°). The following URL was used to obtain the image ID for a specific location: http://api.map.baidu.com/panorama/v2?ak=API_Key&width=600&height=400&location=LAT,LON&pitch=20&fov=90&heading=0/90/180/270. Here, LAT and LON are latitude and longitude, respectively, FOV determines the horizontal field of view of the image, HEADING indicates the compass heading of the camera, PITCH indicates the upward or downward angle of the camera relative to the street view vehicle, and API key is the credential required to validate the request. Each coordinate point was downloaded from four angles to obtain the street view image data, and a total of 442,252 street view images were collected from the Nanshan District.

### 2.3. Restorative perception label data

To generate the restorative perception label data, we arranged the street view image scoring program in a Tencent cloud server such that the experimenter could easily access the server from any computer during the street view restorative perception scoring experiment. In this study, a human-centered perspective was used as an entry point for the evaluation of restorative perceptions. To measure the perception of urban street restoration in the Nanshan District, a total of 25 university students and staff were recruited as volunteers in this study, with a male to female ratio of ~1:1, ranging in age from 21 to 55 years old. Volunteers scored the city street recovery perception of Nanshan District Street view data through a Tencent cloud server. Laumann's Restorative Perception Factor Scale (RCS) (20) was used to guide the volunteers to engage in restorative perception thinking. Among them, the restorative perception questionnaire used in this study was based on the restorative perception factor scale, which is used to investigate people's experience of restorative perception when they observe a street view image, and the final score was the volunteers' true subjective restorative perceptions of the street view. The questionnaire included 50 random street view images and 15 questions corresponding to each street view image (Table 1) to evaluate the perception of street restorability in terms of four aspects, i.e., escape, extent, fascination, and compatibility. We studied the time criteria for scoring single images in other experiments, and we informed the volunteers to observe each image for no < 10 s (21, 22). During this period the volunteers will feel the restorability of the street view images and score their responses on a seven-point Likert scale, with a score of 0–6, where 0 means the description of the question did not match the volunteer's feelings when they observed street view image, and 6 means the description matched the volunteer's feelings. The scores for the 15 questions were then averaged as the final score. Rather than selecting the street view images perceived by volunteers according to the sequential location of adjacent street viewpoints, we used incomplete randomization for street view image extraction such that the distribution of restorative perceived street view locations covered the entire study area comprehensively (17). Finally, the scoring data for all volunteers' perceptions of street view restorability were aggregated as a labeled dataset to train the random forest model. As a result, we were able to create restorative perception labels. This data labeling method is more convenient, efficient, and cost effective than traditional survey methods because it is conducted entirely over the Internet.

**Table 1:** Restorative perception scale.

**ART factor**	**RCS questions**
Escape	Here you can temporarily forget the worries of work and daily life
	Here you can temporarily forget the pressure of other people's demands and expectations
	Here you can forget about your responsibilities and obligations for a while
Extent	Here everything is interconnected
	Here everything is well integrated into the environment
	The surroundings as a whole are coherent
Fascination	Here are many things you want to explore
	Here are many things you feel curiosity
	Here are many things that attract you
	Here's where you want to spend a long time
	You feel immersed in your surroundings
Compatibility	Here you have the opportunity to do things you like
	Here you will be able to solve some of the problems that arise
	You can quickly adapt to the environment
	Here you can satisfy your travel needs

### 2.4. Data of visual elements of urban streets

To explain the mechanisms that may lead to a location being perceived with high or low restorative perception, we introduced visual element data to determine the association of a place with a high degree of human restorative perception. To interpret restorative perception through visual element data, we used semantic image segmentation techniques to calculate the percentage of semantic object elements in each street view image. Semantic scene parsing is an important technique used to understand the perceptions of a given scene. Here, the goal is to segment and recognize object instances in a street view image. Given an input street image, the trained model is used to predict the type of visual elements for each category. We trained a classical SegNet model that has demonstrated good scene segmentation performance, and the training results exhibited an accuracy of 90.83% on the training set and 89.95% on the validation set. This high level of accuracy can facilitate better explanatory work for restorative perception.

The Cityscapes dataset, which contains 34 types of objects in daily life scenes (e.g., sky, roads, cars, and plants), provides rich explanatory variables to explain restorative perception (23); thus, this dataset was selected as the training dataset for the semantic image segmentation model in this study. The Cityscapes dataset is a semantic understanding dataset of urban street scene image. This dataset primarily contains street scene images captured in 50 different German cities (of which 2,975 for train, 500 for val, 1,525 for test, and 19 classes are turned on by default for training in the dataset used for the research).

**Figure 3:**
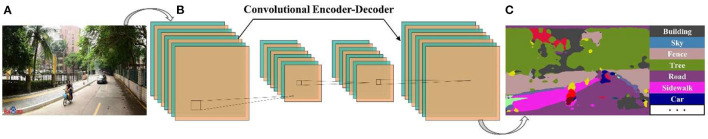
Overview of semantic image segmentation using SegNet trained on Cityscapes dataset: **(A)** input urban natural image; **(B)** SegNet architecture; **(C)** output image with segmentation mask.

To implement and train the deep learning method used in this study and unify the computer training variables, all training processes were executed on the same Windows computer with an NVDIA GeForce GTX 1070 graphics processing unit, an AMD Ryzen5 2600X Six-Core Processor (3.60 GHz), and 16 GB RAM. An overview of the semantic segmentation of the street view images is shown in Figure 3. Here, we applied SegNet to achieve accurate pixel-level segmentation of objects in the target images. SegNet is open-source project for image segmentation developed and published by a team at the University of Cambridge in 2015. The network has two main components, i.e., an encoder and a decoder. The encoder compresses and extracts the object information, the decoder reduces the extracted semantic information to the size of the input image, and each pixel can be classified as its corresponding object information to be represented by its color.

We constructed the SegNet neural network based on the Python language, the Keras deep learning framework, migration learning techniques, and the Tensorboard module to document the training process. For the neural network settings, the input image remapping window was set to 416 × 416 pixels, and the learning rate was set to decrease by 50% when no decrease in loss was observed for three rounds. In addition, early stopping training was set to terminate and finish network training when there no drop in loss was observed for 10 rounds. In the migration learning training phase, the Adam optimizer (24) was used and the learning rate was set to 0.001. In the global learning training phase, the Adam optimizer was used with a learning rate of 0.0001. We used the Keras Tensorboard module to record the training data. Here, the migration learning training phase took 113 min, and the global learning training took 281 min; thus, the total training time was 394 min (6 h and 34 min). The code used to repeat the experiment can be downloaded from our GitHub site (https://github.com/landscapewl/Segnet-Transfer-Learning).

## 3. Methodology

As shown in Figure 1, this study was conducted according to three main processes. Here, we focus on the random forest-based machine learning algorithm used to score the restorative perception of urban streets in the second process to obtain an overall description of the restorative perception of urban streets. In the third process, we used the OLS, GWR, and MGWR models to perform practical exploration of restorative perception and analyze the spatial heterogeneity phenomenon of visual elements and the relationship between visual elements and the restorative perception of urban streets.

### 3.1. Random forests based prediction of urban street restorability perception

To explore urban residents' perceptions of restorative urban streets, we employed a random forest-based machine learning model. This machine learning model has demonstrated good fitness in previous research studies (2, 14, 17). Here, we used it to perform a convenient and effective evaluation of the restorative perception of urban streets in the Nanshan District. Each volunteer scored 50 street images in terms of restorative perception, and the scoring results of all volunteers were used to generate a dataset to train the random forest model. During training, the bootstrap randomly selected two-thirds of the samples for data fitting or classification, and the remaining one-third of the model was defined as out-of-bag (OOB) data, which were used to evaluate the overall model error and the importance of variables. If *X*_*j*_ is input the input variables to the equation, then to calculate the importance of the input variable *X*_*j*_ in the Nth tree *VI*_*n*_, it is necessary to use the sample data extracted by bootstrap to create the regression tree model Tn and calculate the prediction error of OOB again, and finally replace the observations in the variable *X*_*j*_ randomly. Re-establish the model Tn' and calculate the prediction error of OOB'. After processing the prediction error of the two OOB data, the average of all regression tree results is the importance of variable *X*_*j*_ in the Nth random tree, *VI*_*n*_ (*X*_*j*_), by the formula.


$$\begin{array}{l} VI_{n}(X_{j})=\{\sum_{i=1}^{N_{OOB\;}}I[f(X_{i})=f_{n}(X_{i})]\\ -\sum_{i=1}^{N_{OOB\;}}I[f(X_{i})=f_{n}(X_{i}^{'})]\}/N_{OOB} \end{array}$$

### 3.2. Explanatory model for restorative perception of urban streets

After calculating the restorative perception scores of the urban street environments, we examined the association between restorative perception and the visual element features of the urban streets. To understand where and how urban street visual element features affect the perceptions of resilience in space, we used three different regression models, i.e., the OLS, GWR, and MGWR models. Many studies have used OLS as a linear regression method for parameter estimation to explore the association between dependent and independent variables (25, 26). OLS linear regression is a global model that estimates the parameters of the explanatory variables in a linear model by minimizing the sum of the squared differences between the predicted and observed values in the dataset (*a*_*k*_). The formula is as follows.


$$\begin{array}{l} Y_{i}=\alpha_{0}+\overset{m}{\underset{k=1}{\sum }}a_{k}X_{k}+\varepsilon_{i} \end{array}$$

The assumption underlying the use of OLS regression models is that the explanatory variables for restorative perceptions are smooth in space, which suggests that the spatial distance and location between the data will not otherwise affect the final regression results. However, many explanatory variables are in fact non-stationary in space, and the explanatory strength of the data varies across locations in space; thus, the effect of different spatial locations may differ. If Koenker (BP) is statistically significant (*p* < 0.05), which indicates that the study data are spatially non-smooth, then the data should be modeled using a spatial regression model (27). GWR is a spatial regression model first proposed by Fotheringham et al. in 1996. The GWR model can handle the spatial non-stationarity problem in regression analysis effectively (28). Many studies have utilized the GWR model in spatial modeling. For example, Zhou used the GWR model to explore the causes of haze pollution in China, and Robinson used GWR models and geostatistics to improve the accuracy of nitrogen dioxide pollution mapping. In addition, Liu explored spatial variability, temporal variability, and spatial differentiation of confirmed COVID-19 cases in the Hubei Province using a GWR model. If the restorative perceptual data are non-stationary in space, the GWR model can be used as a spatial analysis tool. The GWR is expressed as follows.


$$\begin{array}{l} Y_{i}=\alpha_{0\left(u_{i},v_{i}\right)}+\overset{m}{\underset{k=1}{\sum }}a_{k\left(u_{i},v_{i}\right)}X_{k\left(u_{i},v_{i}\right)}+\varepsilon_{i} \end{array}$$

Here, *Y*_*i*_ is the recovery perception score and its coordinates (*u*_*i*_,*v*_*i*_), α_0(*ui, vi*)_ is the intercept of the model, *a*_*k*(*ui, vi*)_ is the regression coefficient of the *k*^th^ independent variable data at (*u*_*i*_,*v*_*i*_), *X*_*k*(*ui, vi*)_ is to the *k*^th^ attribute at position *i*, and ε_*i*_ is the random error of the model. The regression coefficients of the restorative perceptions obtained using this model can vary in space at different locations, and we can understand how the explanatory variables affect restorative perceptions of urban streets in space according to the variation of the regression coefficients of the explanatory variables in space. To use the GWR model, a neighborhood range must be determined, meaning the data points included in this range. The GWR model uses a single fixed bandwidth to define the neighborhood, which means that all regression coefficients of the explanatory variables are assumed to vary in the same spatial scale. The bandwidth is in distance-based metric units. Using Here, using the bi-square kernel, all data points within the bandwidth range are weighted inversely by the distance to the center of the local region, and the data points outside the bandwidth range are weighted as 0. In this study, we used golden section search optimization with the routine Akaike information criterion (AICc) as the criterion for the model. Both local *R*^2^ and global AICc can be used as parameters to evaluate the model. GWR is performed using the same bandwidth when evaluating the regression coefficients of the explanatory variables.

In fact, the explanatory variables may have different scales of influence in space. The MGWR model allows each explanatory variable to have separate bandwidth, and the size of the bandwidth is scaled according to the scale of the space, which has the potential to reduce errors in parameter estimation (29) using the following equation.


$$\begin{array}{l} Y_{i}=\alpha_{0\left(u_{i},v_{i}\right)}+\overset{m}{\underset{k=1}{\sum }}a_{bwk\left(u_{i},v_{i}\right)}X_{k\left(u_{i},v_{i}\right)}+\varepsilon_{i}\; \end{array}$$

This model is a modification of the GWR model. Here, *a*_*bwk*_ is the *k*^th^ explanatory variable with an added bandwidth term *bw*, and each explanatory variable has a separate spatial scale. MGWR differs from GWR in that it uses an adaptive nearest neighbor bandwidth kernel, which specifies the number of data points that must be included in each local regression model to handle edge effects and non-uniform spatial distributions. As with GWR, a bi-square distance weighting function is used. For parameter optimization, we used the golden mean search optimization procedure using AICc as the model fit criterion. Here, *R*^2^ and AICc were used to evaluate model fitness, and the parameter estimates were mapped out as in GWR.

## 4. Experiments and results

### 4.1. Detection accuracy of random forest algorithms based on restorative factor scales

From the labeled data scored by the volunteers for restorative perception, two-thirds of the data were used to train the random forest model, and the remaining one-third was used to test the accuracy of the model. The fitting results are as follows, with average error of 2.01%, RMSE of 2.91, OOB Error of 5.96%, and OOB RMSE of 8.47 for the city street restorative perception results. The accuracy of estimation of restorative perception of urban streets based on random forest was >90% on average; thus, this model has strong practical application in predicting human perception.

### 4.2. Spatial distribution of restorative perception of urban streets

**Figure 4:**
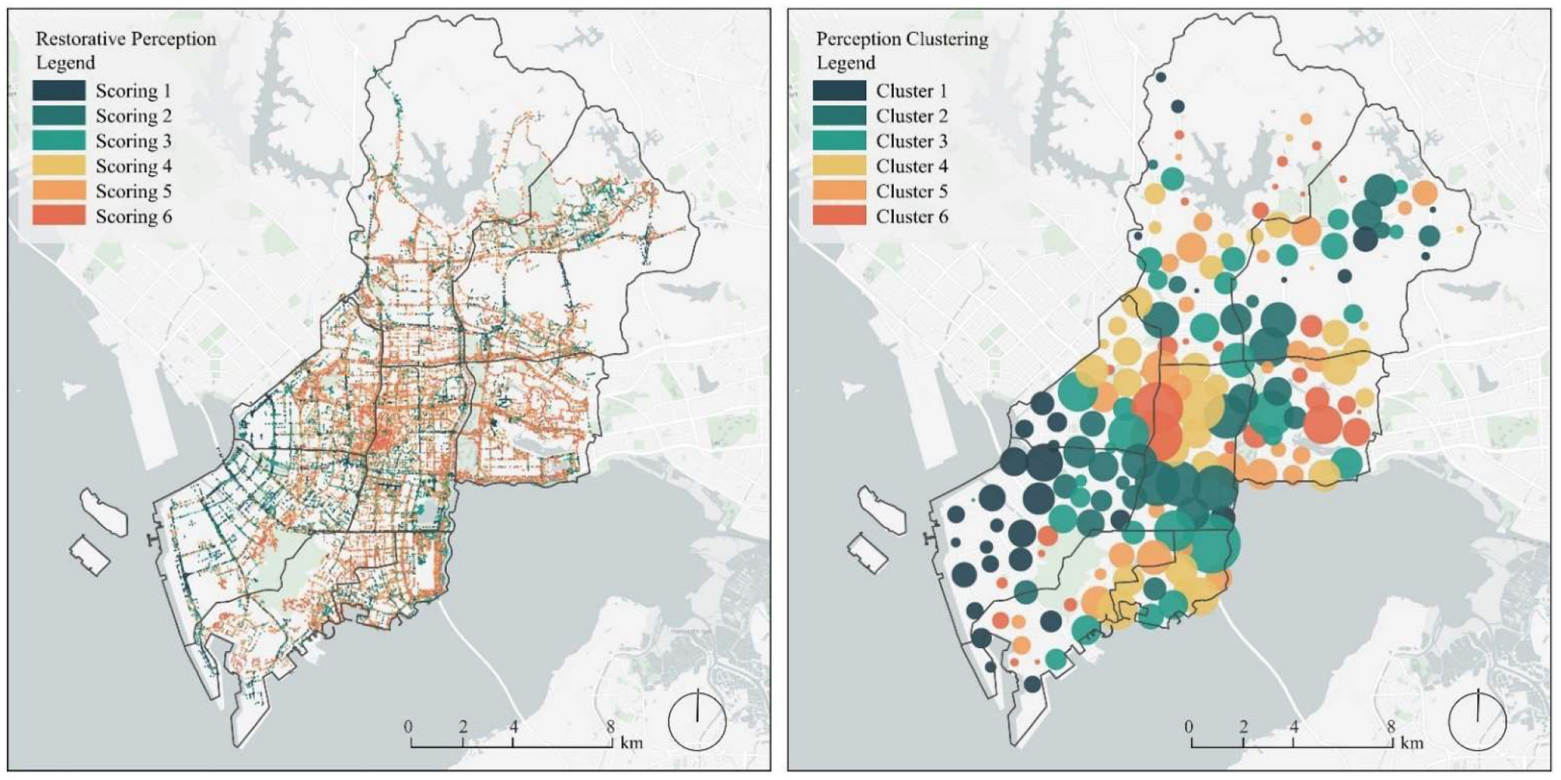
Distribution of restorative perception scores on street **(left)** and restorative perception clustering with different scores **(right)**.

We divided the restorative perception scores for the urban streets in Nanshan District according to the natural breakpoint method, which is divided into six categories. In the legend of Figure 4, left, the restorative perception scores are shown as Scoring 1, Scoring 2, Scoring 3, Scoring 4, Scoring 5, and Scoring 6 in descending order. To observe the distribution of restorative perceptions in the Nanshan District, we also performed cluster analysis of the restorative perceptions. As shown in the legend of Figure 4, right, the clusters of restorative perception scores from low to high are Cluster 1, Cluster 2, Cluster 3, Cluster 4, Cluster 5, and Cluster 6. As can be seen, the restorative perception varies considerably in different regions of the study area. The higher recovery perceptions are more concentrated and primarily distributed in the Yuehai street, Shahe street and Shekou street areas of the Nanshan District, while lower recovery perceptions are more dispersed and primarily distributed in the Nanshan street areas of the Nanshan District. Shenzhen University is located in that area of Nanshan District, with good greenery in the school and a better environment compared to other areas in Nanshan District. This area is more open; thus, there is a higher restorative perception. Other areas in the Yuehai street area of Nanshan District have large urban parks with good plant configurations, garden vignettes, and water features, which helps create a natural and vivid urban landscape. This is an important reason for the high restorative perception of the that area. In contrast, the Nanshan street area region has a low level of greenery, freight terminals, and logistics factories. In addition, a monotonous construction method was used for the main roads in this region, and the skyline is monotonous. Finally, this region offers only a single urban function. These factors likely contribute to the low restorative perception of this region.

**Figure 5:**
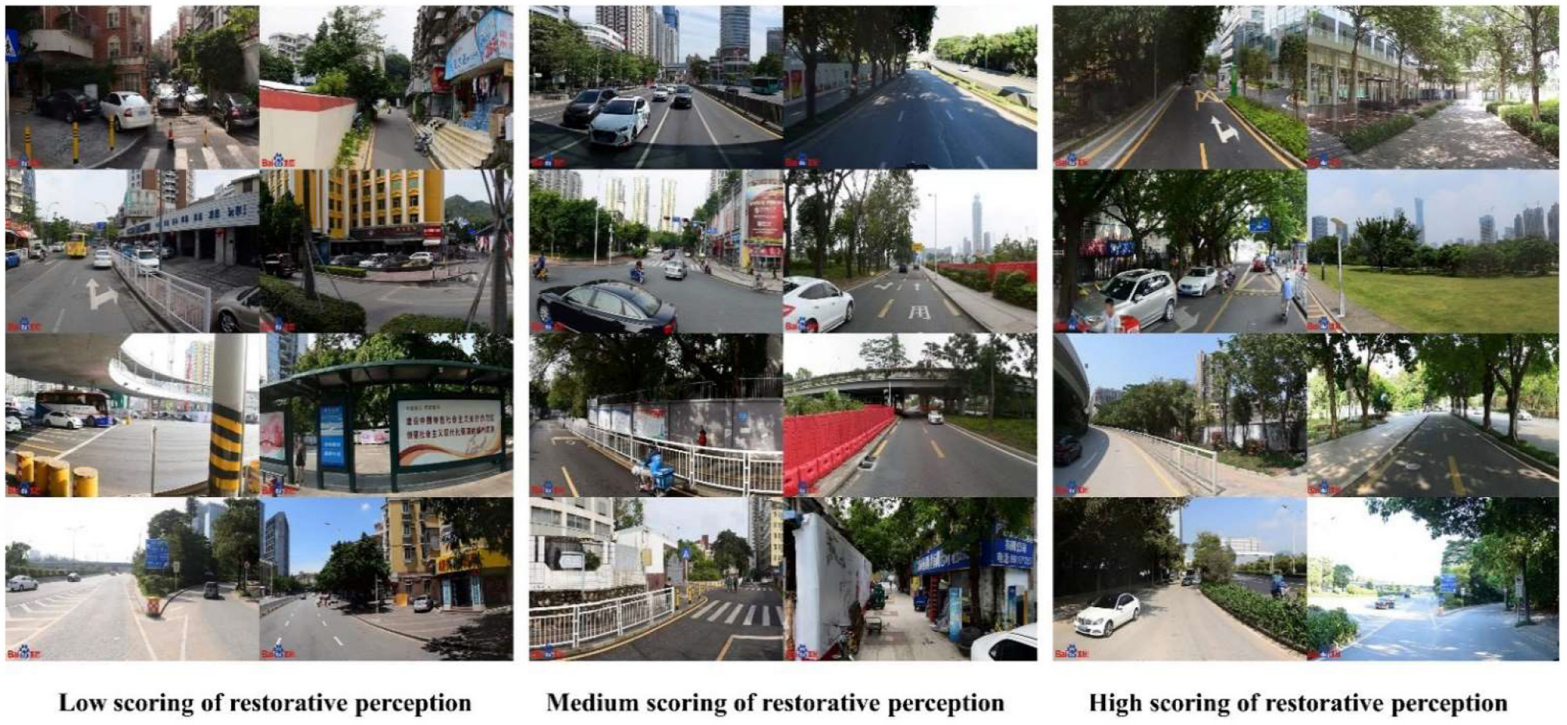
Samples of labeled street view images classified by the machine learning model. Street view images that represent low scoring of restorative perception **(left)**; street view images that represent medium scoring of restorative perception **(middle)**; and street view images that represent low scoring of restorative perception **(right)**.

We classified the street views identified by the machine learning model into three categories according to their scores. Here, street views with restorative perception scores < 2 were defined as low restorative perception street views, those with scores between 2 and 4 were defined as medium restorative perception street views, and those with scores >4 were defined as high restorative perception street views. Figure 5 shows representative street views for low, medium, and high restorative perception scores.

**Figure 6:**
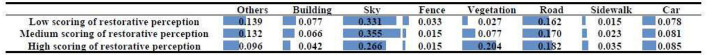
Percentage of visual elements with low, medium, and high perception of urban street restorability.

Table 2 shows the eight visual elements of the street view with the highest average share in the image segmentation process. These visual elements are the most likely to have a positive impact on the perception of restorative urban streets. The visual elements of the streetscape with low, medium and high restorative perception scores were averaged as the visual element percentage characteristics of each street under this perception (Figure 6). We found that the proportion of high restorative perception is low for others and low for building relative to low to medium perception. In addition, the proportion of vegetation is significantly higher than that of low to medium restorative perception. We assume that others may have increased the level of chaos in the street space, resulting in a poor restorative perception. A high proportion of buildings block part of the visual landscape and sunlight, which may lead to a depressed mood in residents and reduce the restorative perception. Plants release oxygen and negative ions through respiration, which regulate the function of the human cerebral cortex and balance the excitatory and inhibitory mechanisms, thereby reducing fatigue, improving mood, and increasing the restorative perception.

**Table 2:** Top eight visual elements identified after segmentation of BSVIs.

**Number**	**Visual elements**	**Mean**	**Max**	**Min**	**S.D**.
1	Others	0.112	0.558	0.001	0.075
2	Building	0.053	0.527	0.001	0.066
3	Sky	0.300	0.714	0.001	0.112
4	Road	0.176	0.441	0.001	0.084
5	Sidewalk	0.029	0.343	0.001	0.035
6	Car	0.083	0.331	0.001	0.045
7	Fence	0.005	0.106	0.001	0.009
8	Vegetation	0.156	0.691	0.001	0.119

### 4.3. Regression results

To understand the causes of reasons in restorative perception, we conducted experiments using the OLS, GWR, and MGWR models. To explore the association between restorative perception of urban streets and different visual elements. First, OLS regression of urban street restorative perception with each visual element of the street was performed. OLS regression can determine whether the study data are smooth in space and is the prior judgment for spatial regression. Table 3 shows that the VIF of each visual element is < 7.5, which indicates that there is no covariance problem. Table 4 shows that the Koenker (BP) is significant, and it is necessary to judge the significance of each visual element by Robust_Pr. The significance *P* are < 0.05, which means that each visual element is statistically significant. However, Koenker (BP) is significant, which proves that each visual element is non-stationary in space, and this indicates that the same variables are fitted differently in different regions of space and that there is heterogeneity; thus, the next step of spatial regression analysis of the GWR and MGWR models can be performed. To explore the most suitable spatial regression model to explain the relationship between restorative perception and each street visual element, we compared the performance of the three models. The results are shown in Table 5. As can be seen, regardless of the model, others, buildings, and fences are negatively correlated with restorative perceptions; thus, these elements are obstacles to enhancing restorative perceptions. Roads, sidewalks, vegetation, and the sky are all positively correlated with restorative perceptions; thus, these elements have a positive impact on enhancing restorative perceptions. GWR and MGWR have significantly improved the model goodness-of-fit (*R*^2^) compared to OLS models. Modeling with spatial heterogeneity in mind can obtain more details of street visual elements and restorative perceptual interpretation. Meanwhile, we can find that MGWR has the lowest AICc among the three models, which is a measure of a model's performance, and a smaller AICc value means a better performance of the model (30). Therefore, we will use MGWR to conduct a study on the relationship between restorative perception and street visual elements of urban streets.

**Table 3:** Summary of OLS results (model variables).

**Variable**	**StdError**	**t-Statistic**	**Probability**	**Robust_SE**	**Robust_t**	**Robust_Pr**	**VIF**
Intercept	0.043	64.774	0.000^***^	0.043	65.721	0.000^***^	–
Others	0.076	−6.450	0.000^***^	0.074	−6.607	0.000^***^	2.083
Building	0.079	−4.318	0.000^***^	0.079	−4.325	0.000^***^	1.813
Sky	0.065	12.450	0.000^***^	0.064	12.574	0.000^***^	3.445
Road	0.074	31.112	0.000^***^	0.075	30.679	0.000^***^	2.550
Sidewalk	0.128	22.033	0.000^***^	0.121	23.352	0.000^***^	1.311
Car	0.077	−4.233	0.000^***^	0.068	−5.656	0.000^***^	3.323
Fence	0.223	−31.612	0.000^***^	0.245	−28.760	0.000^***^	1.560
Vegetation	0.0690	118.287	0.000^***^	0.069	118.639	0.000^***^	3.664

^***^Denotes significance at the 0.1% level, ^**^at the 1% level, and ^*^at the 5% level.

**Table 4:** OLS diagnostics.

**Indicator**	
Number of observations	110,563
Multiple *R*^2^	0.553
Joint *F*-Statistic	7,116.729
Joint Wald Statistic	60,169.649
Koenker (BP)	2,887.57
AICc	115,384
Adjusted *R*^2^	0.552
Joint *F*-Statistic's Prob	0.000^***^
Joint Wald Statistic's Prob	0.000^***^
Koenker(BP)'s Prob	0.000^***^

R^2^ and adjusted R^2^ were nearly identical; thus, only R^2^ is reported.

^***^Denotes significance at the 0.1% level, ^**^at the 1% level, and ^*^at the 5% level.

**Table 5:** Model regression results.

	**OLS coefficients**	**GWR coefficients**		**MGWR coefficients**	
**Variables**	**Mean**	**Mean**	**Min, Max**	**Mean**	**Min, Max**
Intercept	0.000	0.214	(−4.534, 9.189)	0.230	(−1.943, 4.885)
Others	−0.029^**^	−0.069	(−1.293, 1.587)	−0.067	(−0.927, 1.032)
Building	−0.018^**^	−0.106	(−10.847, 10.717)	−0.114	(−2.315, 1.778)
Sky	0.072^**^	0.092	(−6.787, 3.817)	0.179	(−2.941, 2.198)
Road	0.155^**^	0.049	(−1.968, 1.814)	0.056	(−0.925, 0.851)
Sidewalk	0.079^**^	0.031	(−5.572, 3.939)	0.030	(−1.968, 1.285)
Car	0.001^**^	0.201	(−1.346, 0.924)	−0.012	(−0.948, 0.647)
Fence	−0.123^**^	−0.228	(−4.566, 6.597)	−0.218	(−1.273, 1.228)
Vegetation	0.705^**^	0.652	(−3.439, 6.360)	0.651	(−2.451, 3.805)
*R* ^2^	0.552	0.740		0.756	
AICc	115,384.147	78,025.554		73,858.933	

R^2^ and adjusted R^2^ were nearly identical; thus, only R^2^ is reported.

^**^at the 1% level, and ^*^at the 5% level.

### 4.4. Analysis of perception heterogeneity of restorative urban streets

Spatial heterogeneity was observed in the relationship between each visual element and the perception of street restorative, GWR and MGWR regression analyses were conducted to explore the association and patterns between them in more detail. The bandwidth of the variable measures the spatial scale of the action of each process, which can reflect the differences in the perception of urban street restorative according to the different visual street elements. Note that larger action scales indicate weaker spatial heterogeneity in the effect of the influencing factor and stronger spatial heterogeneity. Here, GWR is fixed bandwidth, and MGWR does not have fixed bandwidth. As shown in Table 6, the spatial heterogeneity of the intercept, sky, buildings, and vegetation is high. The differences in the style diversity among buildings, plant species, and growth status in space explain the high heterogeneity of these visual elements in space. In contrast, the spatial heterogeneity of others, fences, roads, sidewalks, and cars is weak. Among these visual elements, cars and sidewalks are near to the global scale, and cars and sidewalks are more consistent in style and have no characteristic distribution in space, which may help explain the low heterogeneity.

**Table 6:** Summary of model bandwidth results.

**Indicator**	**OLS**	**GWR**	**MGWR**
Bandwidth	—	135 (intercept)	62 (intercept)
	—	135 (others)	91 (others)
	—	135 (building)	65 (building)
	—	135 (sky)	55 (sky)
	—	135 (fence)	110 (fence)
	—	135 (vegetation)	75 (vegetation)
	—	135 (road)	116 (road)
	—	135 (sidewalk)	151 (sidewalk)
	—	135 (car)	136 (car)

**Figure 7:**
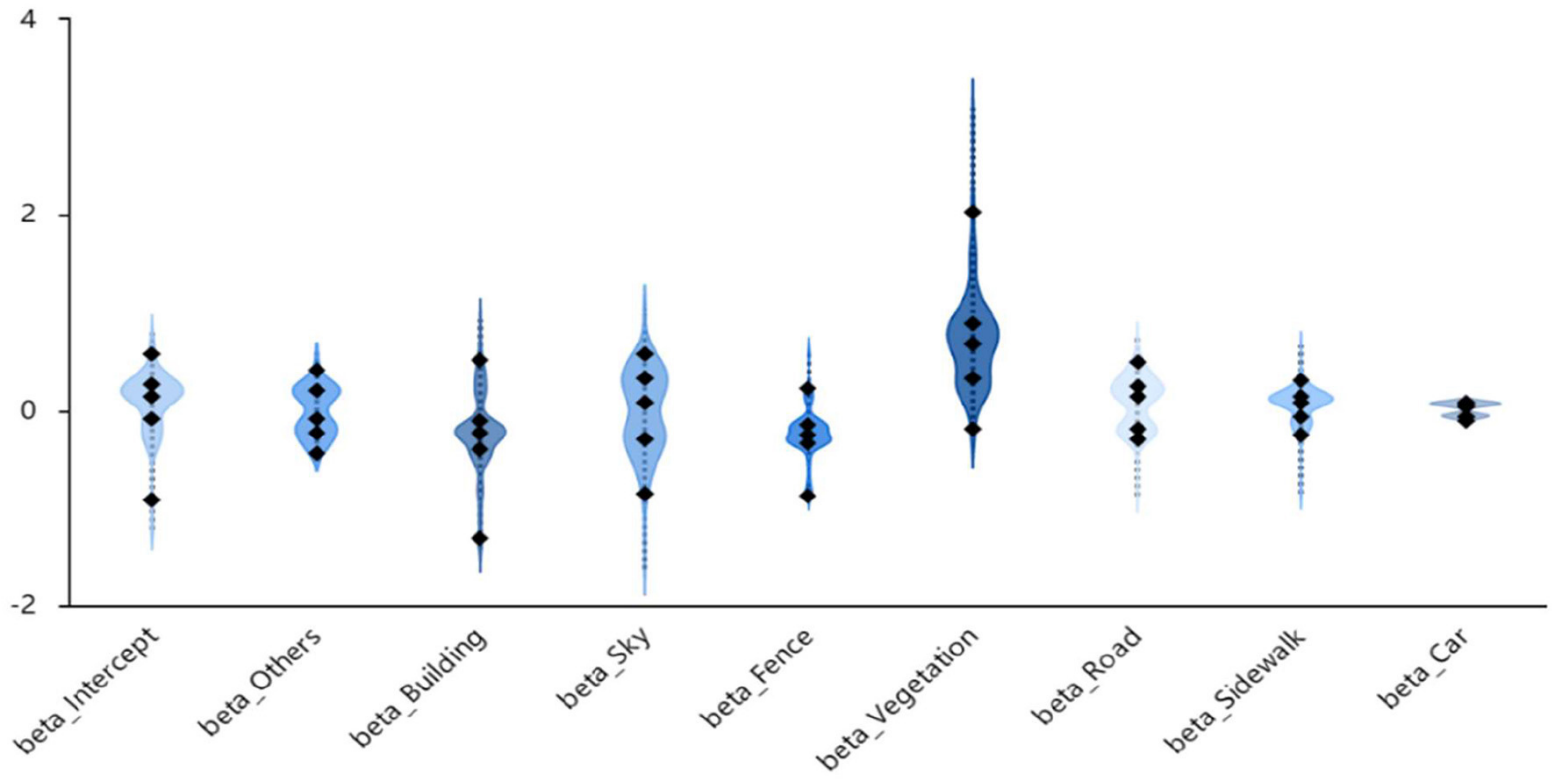
Violin plot of MGWR model regression coefficient distribution.

To obtain a clearer view of the model regression coefficients of the MGWR model, violin plots of the restorative perception regression coefficients were produced by retaining the data with significant (*p* < 0.05) data for each visual element, as shown in Figure 7. Violin plots can be used to show the distribution and density of the data by combining the advantages of box line and kernel density plots (31). The curved line in the violin plot is a symmetric display of the kernel density distribution, the middle part shows the 2.5, 25, 50, 75, and 97.5% quartiles, and the straight line in the middle of the violin plot connects the minimum and maximum values.

The distribution density of coefficients >0 in the Intercept term is much higher than that of the coefficients that are < 0. This indicates that the impact of the constant term on the perception of restorability is more positive, and in a very few areas it has a negative impact. In addition, the impact of different locations on the perception of restorative of the street is more different. The distribution densities of coefficients >0 and < 0 for the others and road terms are approximately equal. This indicates that the positive and negative effects of others on restorative perceptions are approximately the same, and there is little difference in the effects of others on restorative perceptions in different zones. The highest density of the coefficient distribution for the building term is in that are areas < 0, which indicates that buildings have a negative effect on the restorative perception in most areas. In a very few regions, buildings had a higher positive impact and a higher negative impact, showing a more obvious polarization; thus, the role of building varies greatly between regions. The positive and negative effects of sky on the restorative perception were approximately equal, exhibiting quite strong negative effects in a very few areas. For fences, the region with the highest density of the coefficient distribution was ~0, which indicates that the effect of fences on restorative perception is not prominent and has both positive and negative effects on perception in a very small area. The higher density of distribution of the vegetation coefficients in regions >0 indicates more positive effects on restorative perception, and in a few regions the positive effect is quite strong. The region with the highest density of the sidewalk and car coefficient distribution was ~0, which indicates that the effect of sidewalks on restorative perception was not prominent.

The regression results of MGWR for each street point were averaged and projected onto a square hexagon with a side length of 120 m. The hexagonal cell grid shares more adjacent edges and the distance between the centers of mass of adjacent cells is equal, which makes the hexagonal grid more flexible in setting its radius and other parameters to represent the distribution of restorative perception systems with a smoother transition. Moreover, the hexagon can represent the overall situation of each small area, which can effectively avoid the errors caused by individual street points and can guide the planning and renovation of different areas.

**Figure 8:**
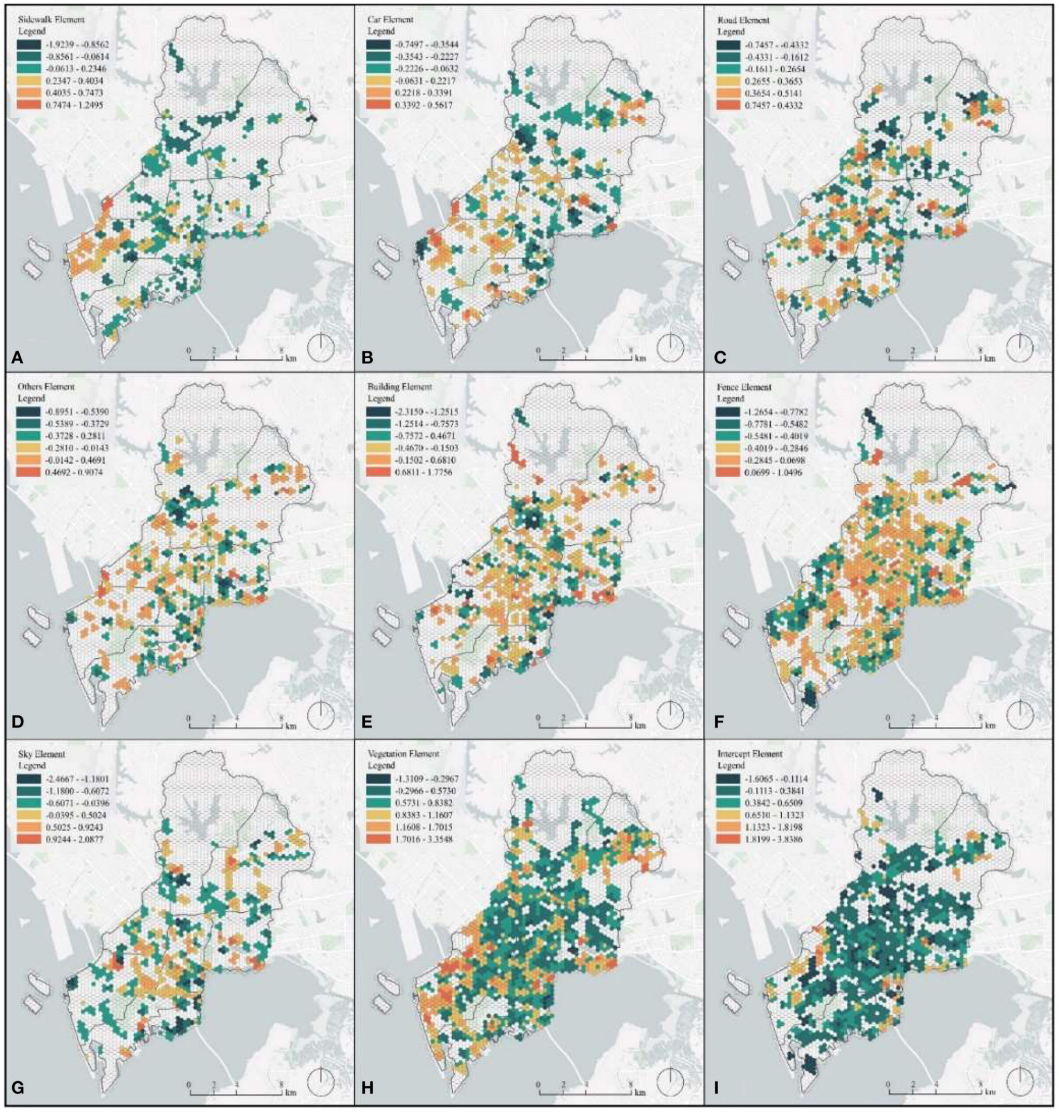
Multiple count MGWR coefficients for Nanshan District: **(A)** sidewalk, **(B)** car, **(C)** road, **(D)** others, **(E)** building, **(F)** fence, **(G)** sky, **(H)** vegetation, and **(I)** intercept.

**Figure 9:**
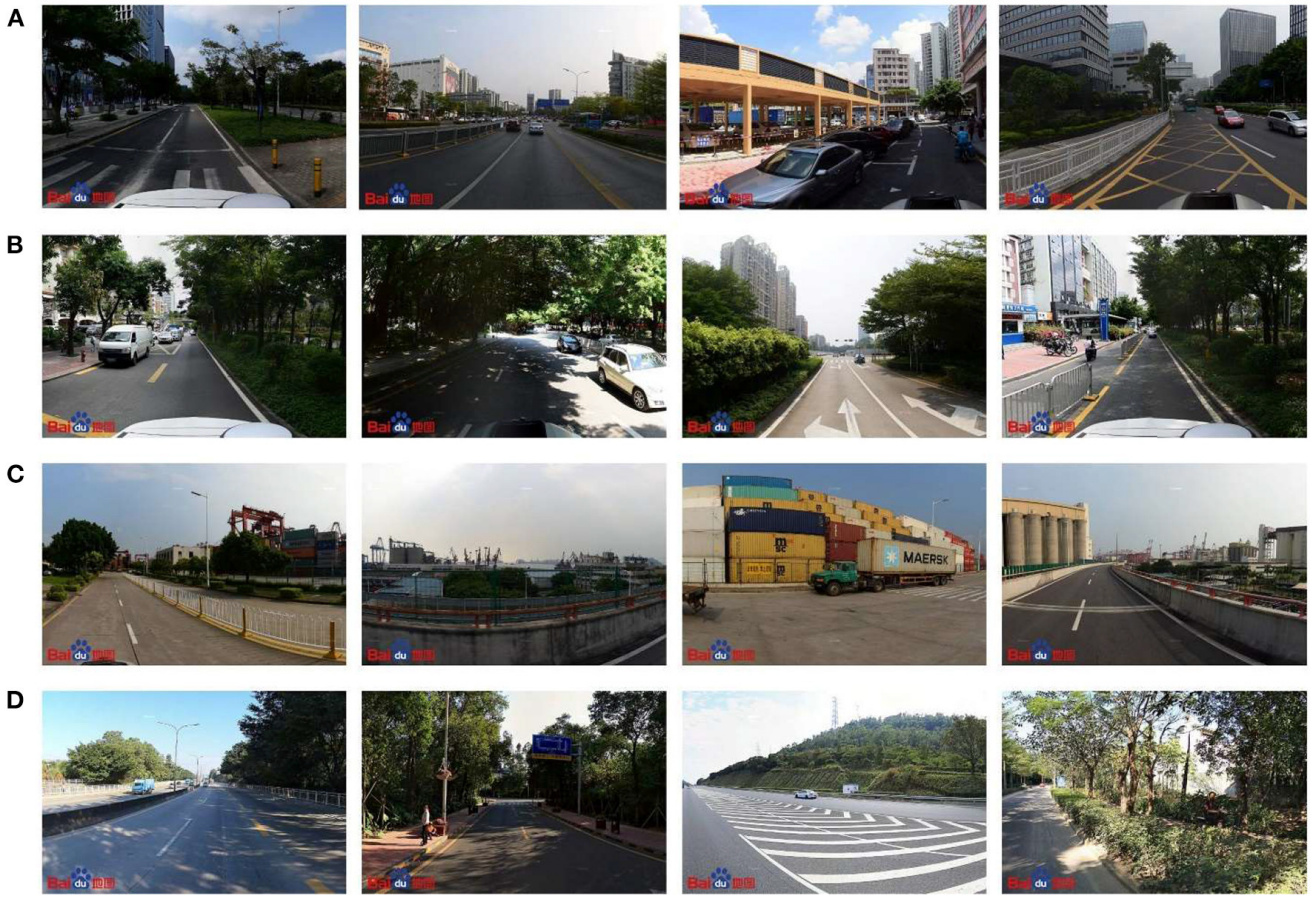
**(A–D)** Example map of a representative area street views.

Figure 8 not only shows the regression coefficients of each visual element, but also the percentage of heterogeneous areas with statistically significant visual elements in the space, and the more areas with colors indicate more areas with significant heterogeneous effects. As shown, the intercept, vegetation, and fence elements are more widespread in the area where heterogeneous effects are evident in Nanshan District. Note that there are obvious regional differences in topography and terrain in the Nanshan District mountainous region. The terrain is roughly high in the north and low in the south. In addition, from north to south, it can be divided into the northern hill basin area, the central low hill terrace area, and the southern low hill flat area, and the terrain decreases from north to south. Differences in topography are possible reasons for the wide distribution of intercept heterogeneity. In most areas, intercept has a positive effect on restorative perception; however, in a few areas, intercept may have a negative effect on restorative perception due to the monotony of the urban built environment. There is a wide variety of vegetation in the Nanshan District, including southern subtropical monsoonal evergreen broad-leaved forests and southern subtropical ravine monsoonal rain forests. The complex structure of vegetation communities, with southern subtropical evergreen high scrub, southern subtropical evergreen low scrub, etc., and the differences in restorative perception brought about by different vegetations may be one reason for the wide range of heterogeneous effects of vegetation in the Nanshan District. Specifically, the coefficient of vegetation is generally high in the Nanshan street area part of Nanshan District, which indicates that vegetation in this region has a high positive correlation with restorative perception, and the increase in vegetation can result in better restorative perception than other regions (Figure 9B). The Nanshan street and Zhaoshang street area part of Nanshan District has a single industrial structure, with more companies in logistics and customs, a single construction approach for urban main roads, and less plant greening compared to other areas of Nanshan District. Thus, the restorative effect of vegetation in that part is higher than in other areas (Figure 9C). Areas where the effect of vegetation heterogeneity is not significant Include Mountain parks with more plant coverage and large areas of homogenized plants without forming distinctive areas; thus, there is no perceived restorative heterogeneity (Figure 9D). Due to the differences in the properties of urban streets, the role played by fences is not exactly the same, which leads to a wider area of significant heterogeneity of fences. In the Yuehai street area, fences showed a positive correlation with restorative perception, and an increase in fences led to increased restorative perception. The Yuehai street and Nantou Street area of Nanshan District is more well developed and has more developed transportation than other areas, and the use of fences may provide a subjective feeling of safety to the urban residents, thereby causing an increase in the perception of restorative (Figure 9A). We also found that the sidewalk, car, road, others, building, and sky elements do not differ due to their distribution characteristics in the urban space, which results in significant regional underrepresentation of heterogeneity. The sidewalk element is positively correlated with restoration perception in the Nanshan Street area part of Nanshan District (a coastal park), and the increase of sidewalks.

## 5. Discussion

### 5.1. Measuring urban restorative perceptions to improve residents' quality of life

The development of global urbanization will cause different degrees of human psychological impacts, and restorative is proposed as a perceptual indicator of city friendliness. Shenzhen is one of the most developed regions in China. Urban commercial land use and boundary expansion are inevitable processes of urban GDP growth in developing countries. However, this is contrary to the subconscious expectations of urban residents for friendly and restorative cities. Thus, it is important to consider the preferences and needs of urban resilience in both current and future development stages. It is also important to consider the perceived measurement and enhancement of urban restorative as an implementable focus of future research. In this study, we attempted to explain the influence of spatial association between urban restorative perception and the visual elements in an urban environment in order to improve the overall urban restorative perception to satisfy the psychological requirements of residents and promote harmonious relationships between residents and the urban. An urban restorative perception measurement framework was developed based on large-scale urban street view data and random forest machine learning algorithms. The MGWR model was used to determine how the urban environment affects residents' perceptions of restorative and the spatial heterogeneity of their impacts. This work has led to relationships between urban planning and development decision makers and mental health researchers. Collaborative research to enhance the perception of urban restorative. Researchers can collect large volumes of data, measure perceptions of urban restorative, and map the perceived restorative clustering of urban residents. This can help urban planners identify areas with high restorative perception clusters. In addition, it will enable researchers to provide solutions to enhance the perceived restorative capacity of urban residents and build more humble urban areas from the perspective of different disciplines and industries.

### 5.2. Measuring perceptions of urban restoration to provide advice to urban planners

Most previous studies have explored the relationship between urban street restorative perception and explanatory variables from the perspective of the entire study area, but the relationship between the two at different spatial locations is unknown, and therefore the information provided to urban planners is limited. In this study, a spatial regression model was used to explore the relationship between restorative perceptions and visual elements on urban streets, not only to understand the role of each visual element at the macro level, but also to grasp the relationship between each visual element and restorative perceptions at the micro level in specific locations. Specifically, visual elements with high heterogeneity should be given priority attention by urban planners, and the effects of these visual elements on restorative perception may vary greatly in different locations. In places with high regression coefficients, the increase of visual elements may bring higher restorative perception effects. Where the regression coefficient is low, the configuration of visual elements is not sensitive to fluctuations in restorative perception and can be planned uniformly. Places with regression coefficients < 0, where visual elements have a negative relationship with restorative perception, should be appropriately reduced in the allocation of visual elements. The scheme of this study can provide urban planners with more detailed advice on the configuration of visual elements in urban streets in urban physical examination or urban micro-renewal.

### 5.3. Exploring spatial heterogeneity in urban restorative perception using MGWR model

We constructed a complete methodological framework using GSV data and machine learning techniques to measure the level of restorative perception in urban spaces. The superior performance and reliability of the MGWR model compared to the OLS and GWR models were verified in our analysis of the spatial heterogeneity of restorative perception. The proposed framework in this study is more streamlined and effective compared to the traditional approach. This was followed by an empirical case study of restorative perception mapping in the Nanshan District of Shenzhen, China. We found that residents' perception of restorative was positively correlated with the percentage of visual road, sidewalk, vegetation, and sky elements, and was negatively correlated with the percentage of visual elements of others, building, and fence elements. In a comparative analysis of the collected data, the AICc values of the GWR and MGWR models were much less than that of the OLS model, and the performance of the GWR and MGWR models was much better than that of the OLS model. We found that the MGWR model outperformed the GWR model in terms of goodness of fit and performance, and the spatial heterogeneity results analyzed using the MGWR model were more scientific and reasonable. We found that the spatial heterogeneity of the intercept, sky, building, and vehicle elements is stronger, and the spatial heterogeneity of the others, fence, road, sidewalk, and car elements is weaker, among which cars and sidewalks are close to the global scale, cars and sidewalks are more consistent in style, and their distribution in space is not characteristic. Thus, we believe that our findings provide practical and innovative contributions. These demonstrate that the visual attributes of an environment have spatially heterogeneous effects on restorative perception, and that big data can be used to improve understanding of the relationship between restorative perception and urban environments.

### 5.4. The novelty of using street view big data with spatial regression models

In previous studies on restorative perceptions, most scholars have started by designing restorative perception questionnaires and conducting questionnaires on a small group of volunteers. The survey results may be more accurate, but the small sample size, time-consuming survey, and small survey scope have limited the research on restorative perceptions. With the development of various disciplines and technology, VR technology and the popularity of various types of big data have provided strong support for the study of restorative perception, but most scholars have used multiple linear regression for the interpretation of restorative perception data, without comprehensive consideration of the spatial heterogeneity of the data. In this study, SegNet is used to segment the visual elements of the Street view, and a random forest model is used to predict the restorative perception scores of urban streets. The process of interpreting restorative perception first utilizes OLS to determine whether the restorative perception data are smooth in space, and then to determine whether a spatial regression model is needed. Secondly, two spatial regression models, GWR and MGWR, were used to compare the explanatory strength of restorative perception, and the MGWR model with the highest explanatory strength was used to explore the connection between restorative perception and visual elements, and to identify the role of each element on restorative perception and the heterogeneous effect in space. This study combines deep learning techniques with spatial regression model, which means that it breaks through the limitations of the traditional approach, expands the scope of the study, reduces the cost of investigation, and increases the sample size of the survey, and also treats the restorative perception using spatial regression model with higher explanatory strength than the traditional single multiple linear regression, which makes the study tends to be refined and accurate. This provides a strong help for urban planners and research scholars to conduct research on the restorative perception of urban streets.

### 5.5. Limitations and future work

Several limitations that will be addressed in future work should be identified and discussed. First, data deviation is a common concern in such studies when perceptual data are collected from cloud servers based on the restorative perception factor scale. We found differences in the classification results of the street view restorative perception in the same part of the city by different volunteers. Here, we used machine learning methods to predict the global perception by excluding parts with restorative perception differences and rather than using data without differences. However, what is more meaningful and exploitable are regions with perceptual differences, or the classification results of multiple acquisitions to obtain the average of the recovered perceptions for the same region. In addition, Shenzhen is one of the most developed cities in China with the highest mix of urban functions; thus, the validity of the results of this study have not been investigated in other cities in China or other parts of the world. Thus, the generalizability of our findings must be investigated using additional research data for other regions. Finally, the impact on the perception of urban restorative is a multifaceted process that influenced by multiple factors. Thus, various other factors that influence perceived urban restorative properties should be further discussed. The future combines, for example, the application of the conclusions of Cervero et al. who concluded that density, diversity, and design are the three basic metrics for assessing 3Ds in street environments (32). To measure the relationship between the urban environment and the restorative perception more comprehensively. It can be used to investigate different methods to enhance the perceived urban restorative properties from multiple perspectives. POI-based facility distribution, spatial distribution data of transit stops should also be added to add to the discussion, and these approaches can be used to investigate ways to enhance perceived urban restoration from multiple perspectives.

## 6. Conclusion

The restorative properties of urban streets have a positive effect on the development and wellbeing of society and are required to facilitate the healthy and virtuous development of society. In this study, we systematically evaluated the perception of urban street restoration using a framework using street view big data, deep learning, and multiple regression models. Specifically, we performed semantic segmentation of the street view images using a deep learning method, and we evaluated the restorative perception of streets in the Nanshan District using a random forest algorithm based on the restorative factor scale. Finally, regression analysis was performed using the OLS, GWR, and MGWR models to explore the correlation between restorative perception and visual street elements in an urban space in detail. This method is more logical in terms of interpretation because it considers the possible spatial heterogeneity effect of the data. We suggest that this methodology can be used as a foundation for urban redevelopment to improve the overall recovery of the city. In addition, we believe that our findings will have important practical implications for the urban street reconstruction and urban computational science fields. The analysis of different regression models in this study showed that the *R*^2^ of MGWR is 0.756, the *R*^2^ of GWR is 0.740, and the *R*^2^ of OLS is 0.552. The MGWR model is more suitable than the OLS and GWR models in exploring the association between perceptions of urban street restorability and urban visual elements. The exploration of different models revealed the existence of the spatial heterogeneity of visual elements on street restorative perceptions by each street. Among them, we found that vegetation, roads, the sky, and sidewalks exhibited a positive relationship with restorative perception. In addition, we found that the other elements, such as buildings, fences, and cars, demonstrated a negative relationship with restorative perception. They can be combined with spatial heterogeneity for comprehensive treatment in future urban transformation processes. We believe that this innovative approach can support the reconstruction of the built environment on the street by linking restorative perception studies with new data and emerging technologies. We expect that this will have important implications for the study of restorative perception in urban environments.

## Data availability statement

The datasets presented in this study can be found in online repositories. The names of the repository/repositories and accession number(s) can be found in the article/supplementary material.

## Author contributions

XH and LW: conceptualization and writing—original draft. XH: resources. JH and TJ: supervision. LW: validation. All authors contributed to the article and approved the submitted version.
